# Acute and preventive treatment of menstrual migraine: a meta-analysis

**DOI:** 10.1186/s10194-024-01848-6

**Published:** 2024-09-04

**Authors:** Cindy Ciat-Wuah Khoo, Chan-Cheng Liu, Michael Lu, Yu-Chen Huang, Hsing-Yu Weng

**Affiliations:** 1 Department of Neurology, Wan Fang Hospital, Taipei Medical University, Taipei, Taiwan; 2 Department of Dermatology, Wan Fang Hospital, Taipei Medical University, Taipei, Taiwan; 3 Department of Neurology, School of Medicine, College of Medicine, Taipei Medical University, Taipie, Taiwan

**Keywords:** Menstrual migraine, Acute treatment, Preventive treatment, Meta-analysis, Triptans, CGRP monoclonal antibody

## Abstract

**Background and objectives:**

About a quarter of migraine cases among women have menstrual migraine (MM), which is usually more severe, longer lasting, and less responsive to treatment than typical migraine. Randomized controlled trials (RCTs) have evaluated the efficacy of several medication in the acute and preventive treatment of MM; this meta-analysis compared the effectiveness of these treatments.

**Methods:**

We conducted systematic searches in the Cochrane Central Register of Controlled Trials, MEDLINE, and Embase databases. The primary outcomes of acute treatment trials were pain relief at 2 and 24 h after treatment compared with placebo or another treatment. The three endpoints we checked for studying MM prevention were: no recurrence of headaches each month, a 50% reduction in monthly migraine days from baseline, and a decrease in the mean number of headache days per month.

**Results:**

Out of 342 studies, 26 RCTs met the criteria. Triptans, combined with or without other analgesics, were superior to placebo in providing pain relief in the acute treatment and prevention of MM. Among the treatments, sumatriptan and lasmiditan demonstrated superior pain relief at 2 h (OR: 4.62) and 24 h (OR: 4.81). Frovatriptan exhibited effectiveness in preventing headache recurrence, whereas galcanezumab and erenumab displayed significant preventive benefits in reducing headache days per month.

**Conclusion:**

Sumatriptan and lasmiditan are effective first-line treatments for acute MM. For prevention, frovatriptan may be the more effective of triptans. Compared with triptans, CGRP monoclonal antibodies, here including erenumab and galcanezumab, are more effective in reducing headache days, and therefore, in preventing MM.

**Supplementary Information:**

The online version contains supplementary material available at 10.1186/s10194-024-01848-6.

## Introduction

Migraine is a primary headache disorder that the one-year prevalence of women is about three times more than that of men (17.1% vs 5.6%) [1]. Menstrual migraine (MM), a type of migraine associated with the menstrual cycle, affects 4% to 8% of women in their reproductive years. This type of migraine is more severe, longer lasting, and less responsive to treatment than typical migraine [2, 3]. The International Classification of Headache Disorders, 3rd edition (ICHD-3) [3] classifies MM into 2 subtypes: pure MM (PMM), which occurs during menstruation or from 2 days before to the third days of menstruation, and menstrual-related migraine (MRM), which occurs both during menstruation and at other times in the menstrual cycle.

Triptans are selective serotonin (5-hydroxytrhyptamine, 5-HT) 1B/1D receptor agonists that disrupt the mechanism of migraines by inducing intracranial vasoconstriction and inhibiting vasoactive neuropeptide release [4]. In their systematic review, Nierenburg et al. demonstrated that triptans may be particularly effective for the acute treatment and short-term prevention of MM [5]. Two meta-analysis in 2013 and 2023 reported that 2 triptans, frovatriptan and zolmitriptan, were both more effective than placebo in reducing the need for rescue medication and the incidence of MM per perimenstrual period. Frovatriptan was associated with a lower likelihood of adverse events than zolmitriptan [6, 7].

Calcitonin gene-related peptide (CGRP) plays a pivotal role in migraine headaches. Recent advancements have introduced medications aimed at inhibiting CGRP or its receptors, thereby offering an innovative treatment option for migraine prevention and treatment. CGRP treatment is divided into 2 types: CGRP monoclonal antibodies, such as erenumab, fremanezumab, and galcanezumab, and CGRP receptor antagonists. CGRP monoclonal antibodies are administered monthly or quarterly, and they act by blocking the CGRP receptor, thereby reducing the frequency and severity of migraines [8].

Lasmiditan is a novel class of medication that acts as a serotonin receptor agonist with high affinity for the selective 5-HT1F receptor, specifically designed for the acute treatment of migraines. Unlike other triptans, lasmiditan does not induce blood vessel constriction, making it a suitable option for patients who cannot tolerate triptans due to cardiovascular concerns [9].

Several studies have demonstrated the effectiveness of such medications in treating MM. The present meta-analysis examined both existing and new medications for MM, comparing their effectiveness in acute and preventive treatments. The goal of this meta-analysis was to determine differences in efficacy to provide more effective treatment recommendations.

## Methods

This meta-analysis adhered to the Preferred Reporting Items for Systemic Reviews and Meta-analysis (PRISMA) guidelines (Supplementary Table 1). The study protocol was registered with PROSPERO (CRD42023452723).

### Data source and search strategy

We searched the Cochrane Central Register of Controlled Trials, MEDLINE, and Embase databases by using the search terms “menstrual”, “migraine”, “prevention”, and “treatment”. We included studies published on or before September 15, 2023, in our analysis. All selected studies were human clinical trials and published in English.

### Study selection and eligibility criteria

Studies were included in our analysis if they (1) included patients diagnosed as having MM in accordance with the International Headache Society or the International Classification of Headache Disorders, Second Edition (ICHD-2) [10], (2) treated MM during the menstrual window (day − 3 to day + 3 of menstruation) using triptans, (3) assessed pain relief at 2 or 24 h, (4) documented headache days or recurrence after prevention treatment, and (5) were randomized controlled trials. Two authors independently screened the studies on the basis of the inclusion criteria, and any disagreements between the 2 reviewers were resolved through discussion with a third author until consensus was reached. Studies were excluded if they (1) involved animals, (2) had a title or abstract irrelevant to the focus of our study, (3) were not RCTs, or (4) were RCTs with a high risk of bias.

### Outcomes

The first primary outcome was pain relief at 2 h after medication use for treating a single acute MM attack. The second primary outcome was pain relief 24 h after medication use for the same. A surface under the cumulative ranking analysis (SUCRA) was performed to identify treatments with the highest probability of efficacy for MM.

Three outcome endpoints for MM were analyzed: (1) the percentage of participants experiencing no headache recurrence every month during the trial, (2) the percentage of participants achieving a ≥ 50% reduction from baseline in monthly migraine days, and (3) a decrease in the mean number of headache days per month.

### Quality assessment

**Fig. 1:**
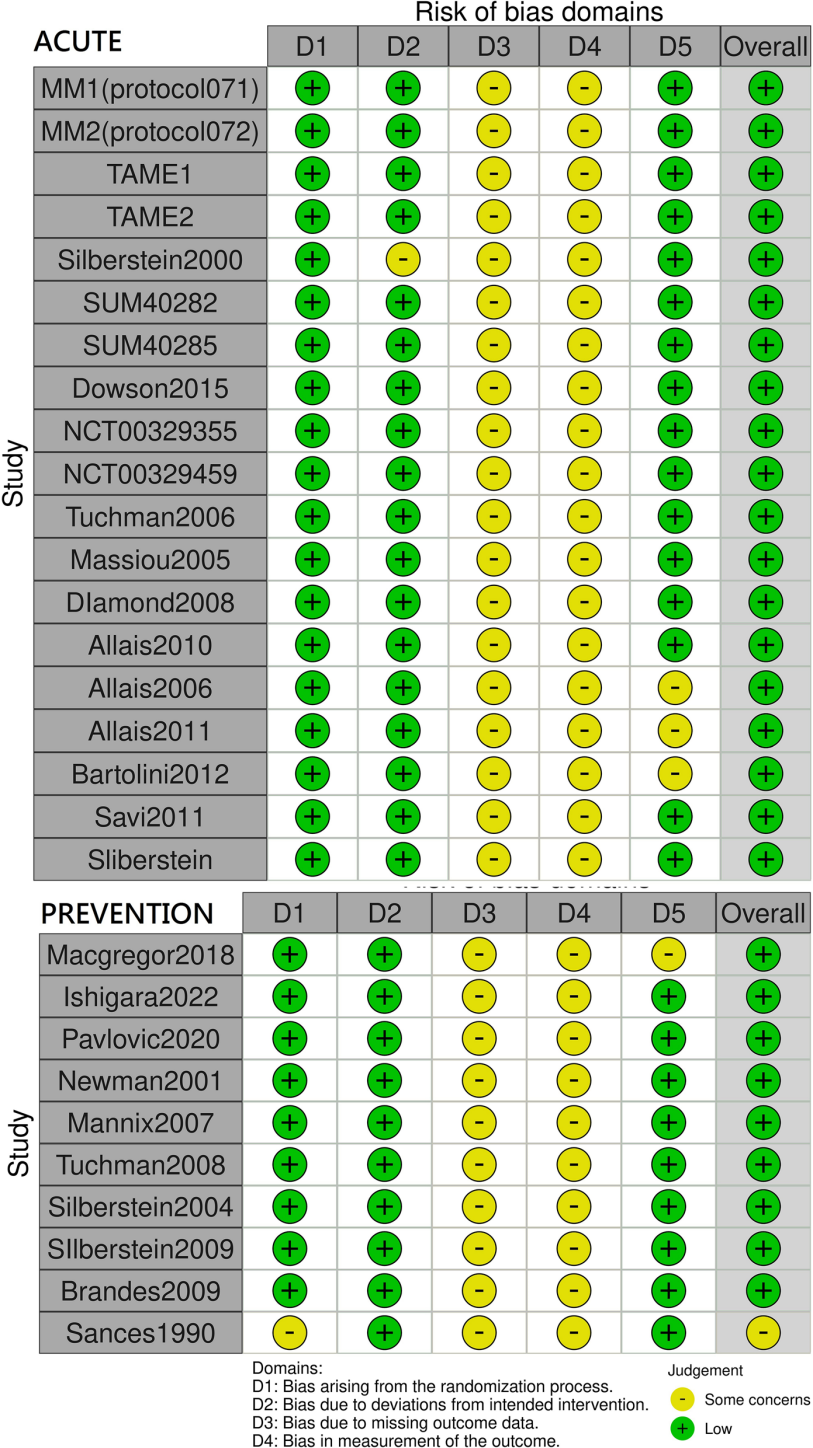
Risk of bias in studies

Study quality was assessed using the Cochrane Collaboration’s risk of bias tool and the PRISMA checklist. Two reviewers initially evaluated the risk of bias for each included RCT (Fig. 1), and any disagreements between them were resolved through discussion, adjudicated by third and fourth reviewers. The risk of bias assessment covered the following domains: random sequence generation, allocation concealment, blinding of participants and personnel, blinding of outcome assessment, incomplete outcome data, and selective reporting. This process involved assigning a judgment of “low risk” of bias (green dot), “high risk” of bias (red dot), or “unclear risk” of bias (yellow dot) to each domain.

### Data extraction

The following information was extracted from the included studies and entered into a computer spreadsheet: clinical trial number/name, study period, sample size, age, treatment regimen, and outcome assessment (Tables 1 and 2). Various scales were used to determine the extent of pain relief provided by the medication or the satisfaction of participants with the medication. Multiple 4-point pain grading scales were used in different trials; one trial asked respondents to report changes in baseline pain from severe to moderate, mild, or no pain. In prevention trials, treatment effects were evaluated on the basis of the number of migraine days per month. Three types of endpoints were included in prevention trials: the number of cases with no headache recurrence, a 50% reduction in headache days, and the decrease in the mean number of headache days after prevention treatment.

**Table 1:** Characteristics of randomized controlled trials on acute migraine treatment included in the present study

Trial	Treatment	Total n(m;c)	Classified by ICHD criteria?
Mannix2007(MM1) [11]^a^	Rizatriptan10mg	357(234;157)	Yes
Mannix2007(MM2) [11]^a^	Rizatriptan10mg	348(245;103)	Yes
Martin2007(TAME1) [12]^a^	Rizatriptan10mg	43(34;9)	Yes
Martin2007(TAME2) [12]^a^	Rizatriptan10mg	51(29;22)	Yes
Silberstein2000 [13]^a^	Rizatriptan5mg,10 mg	910(534;376)	Yes
Landy2004(SUM40282) [14]	Sumatriptan50mg,100 mg	265(133;132)	No
Landy2004(SUM40285) [14]	Sumatriptan50mg,100 mg	233(115;118)	No
Dowson2005 [15]	Sumatriptan100mg	78(39;39)	No
Cady2011(NCT00329355) [16]	Sumatriptan85mg + Naproxen500mg	262(124;138)	Yes
Cady2011(NCT00329355) [16]	Sumatriptan85mg + Naproxen500mg	252(124;128)	Yes
Tuchman2006 [17]	Zolmitriptan2.5 mg	334(174;160)	No
Massiou2005 [18]	Naratriptan2.5 mg	229(115;114)	No
Diamond2008 [19]^a^	Almotriptan12.5 mg	42(20;22)	No
Allais2010 [20]	Almotriptan12.5 mg	244(122;122)	No
Allais2006 [21]	Almotriptan12.5 mg vs. Zolmitriptan2.5 mg	255(Almotriptan136;Zolmitriptan119)	No
Allais2011 [22]	Frovatriptan2.5 mg vs. Zolmitriptan2.5 mg	138(Frovatriptan73;Zolmitriptan65)	Yes
Bartolini2012 [23]	Frovatriptan2.5 mg vs. Almotriptan12.5 mg	148(Frovatriptan79;Almotriptan79)	No
Savi2011 [24]	Frovatriptan2.5 mg vs. Rizatriptan10mg	107(Frovatriptan48;Rizatriptan59)	No
Macgrogor2022 [25]^a^	Lasmiditan100mg	169(90;79)	Yes
Silberstein1999 [26]	AAC(acetaminophen250mg + caffeine85mg + acetylsalicylic acid250mg)	180(85; 100)	No

**Table 2:** Characteristics of randomized controlled trials on menstrual migraine prevention treatment included in the present study

Trial	Treatment	Total n(m;c)	Trial length	Treatment lenth
Macgregor2018 (EVOLVE1 + 2)[27]^a^	Galcanezumab120mg	450(140;310)	6 months	Once per month
Macgregor2018 (CONQUER)[27]^a^	Galcanezumab120mg	348(245;103)	6 months	Once per month
Igarashi2022[28]^a^	Galcanezumab120mg	345(115;230)	6 months	Once per month
Pavlovic2020[29]^a^	Erenumab70mg	151(68;83)	6 months	Once per month
Newman2001 [30]	Naratriptan2.5 mg QD	136(70;66)	1 week per month for 3 months	-2 ~ + 5
Mannix2007 [31]	Naratriptan1mg BID	287(149;138)	6 days per month for 4 months	-2 ~ + 4
Tuchman2008 [32]	Zolmitriptan2.5 mg BID Zolmitriptan2.5 mg TID	161(80;81)	1 week per month for 3 months	-2 ~ + 5
Silberstein2004 [33]	Frovatriptan2.5 mg QD Frovatriptan2.5 mg BID	1507(505;501;501)	6 days per month for 3 months	-2 ~ + 4
Silberstein2009 [34]	Frovatriptan2.5 mg QD Frovatriptan2.5 mg BID	319(161;158)	6 days per month for 3 months	-2 ~ + 4
Brande2009 [35]	Frovatriptan2.5 mg QDFrovatriptan2.5 mg BID	529(177;175;187)	6 days per month for 4 months	-2 ~ + 4
Sances1990 [36]	Naproxen550mg BID	35(18;17)	14 days per month for 3 months	-7 ~ + 6

^a^Post-hoc analysis studies data extract from former studies data, not designed for menstrual migraine

### Statistical analysis

Forest plots were generated to illustrate the 2- and 24-h efficacy of each treatment relative to placebo or other treatments along with 3 outcome endpoints for MM prevention. The effects of the associations are expressed as odds ratios (ORs) with corresponding 95% confidence intervals (CIs). Pooled ORs were estimated using fixed effects. The reduction in monthly migraine days was calculated to evaluate the effect on the mean number of headache days per month. Heterogeneity between studies was tested using the I2 statistic. A network meta-analysis model was also generated to evaluate the strength of the association between each of the triptans and placebos in RCTs. SUCRA was performed to identify treatments with the highest probability of efficacy for treating MM at 2 and 24 h. Statistical analyses were performed using Microsoft Excel, MetaXL, Review Manager, and OpenMeta [Analyst] programs.

## Results

### Study selection

**Fig. 2:**
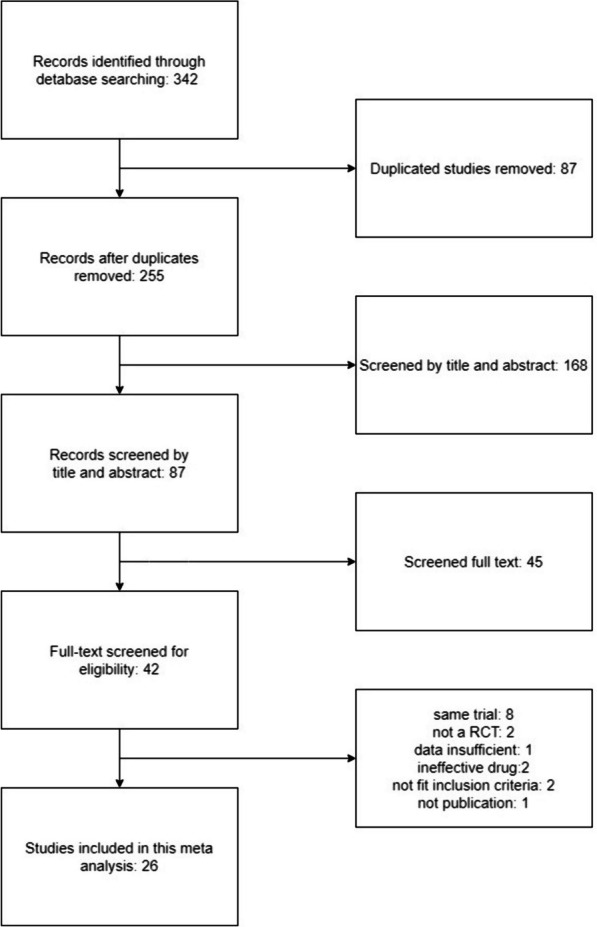
PRISMA flow diagram for study selection

A total of 342 studies were initially obtained through database searches. We removed 87 duplicate studies and 168 studies that did not meet the inclusion criteria. We screened the full texts of the remaining 87 studies and excluded 45 of them. Then 16 studies were excluded because they extracted data from the same trial (*n* = 8), non-RCTs (*n* = 2), trials with insufficient study data (*n* = 1), trials with ineffective drugs (*n* = 2), trials with endpoints that did not fit the inclusion criteria (*n* = 2), or trials not yet published (*n* = 1). In total, 26 studies were included in the systematic review and meta-analysis (Fig. 2).

### Study characteristics

Tables 1 and 2 [11–36] present an overview of participants included in RCTs that investigated acute or preventive migraine treatment, respectively. This meta-analysis reported data from 8926 patients, with each RCT enrolling 35 to 1507 patients. Nine RCTs did not disclose the age of participants. In the remaining 17 trials, the average age of participants ranged from 34 to 43 years. In the acute treatment for MM, 6 types of triptans were compared with placebos. A 10-mg daily dose of rizatriptan was tested in 3 trials [11–13]; sumatriptan was tested using a 50-mg dose in one trial [15], a 100-mg dose in 2 trials [14, 15], and an 85-mg dose combined with a 500-mg dose of naproxen in one trial [16]. A 2.5-mg daily dose of zolmitriptan and naratriptan were each tested in separate trials, and a 12.5-mg dose of almotriptan was tested in one trial [22]. In an RCT by Cady published in 2011, a combination of sumatriptan and naproxen was compared with a placebo. A trial was conducted using 100 mg of lasmiditan [25]. One study tested a combination of acetaminophen, caffeine, and acetylsalicyclic acid (AAC)[26]. Four studies performed the following binary comparisons: almotriptan at 12.5 mg compared with zolmitriptan at 2.5 mg [21]; frovatriptan at 2.5 mg compared with zolmitriptan at 2.5 mg [22], frovatriptan at 2.5 mg compared with almotriptan at 12.5 mg [23], and frovatriptan at 2.5 mg compared with rizatriptan at 10 mg [24]. For the preventive treatment of MM, 3 types of triptans were compared with placebos. Naratriptan at 2.5 mg once daily and 1 mg twice daily were tested independently [30, 31]. Frovatriptan at 2.5 mg once daily and 2.5 mg twice daily were compared in 3 trials [33–35]. Each trial compared zolmitriptan at 2.5 mg twice daily, zolmitriptan 2.5 mg thrice daily [32], naproxen at 550 mg twice daily [36], and erenumab at 70 mg with placebos [29]. Furthermore, galcanezumab at 120 mg was studied in 2 trials [27, 28].

Nineteen studies included patients both with and without pre-MM aura. Five studies examined only MM without aura. Silberstein (2000) and Allais (2006) included no information on the aura. Ten of the included trials diagnosed MM in accordance with the ICHD criteria, whereas the other 16 trials diagnosed MM based on the IHS diagnostic criteria. Thirteen trials evaluated migraines with pain grading classified into absent, mild, moderate, and severe pain. For migraine evaluation, Cady (2011) used a satisfaction grading scale (0 to 7). The included trials required patients not to take other medicines for at least 2 weeks prior to participation with the exception of the 5 following trials: Martin (2007; TAME1), Martin (2007; TAME2), Silberstein (2000), Allais (2006), and Allais (2011). Trials involving the use of galcanezumab and erenumab were conducted over 6 months, and these 2 drugs were administered subcutaneously once a month. Table 2 provides information regarding the duration of use and length of trials for other medications used for MM.

The efficacy of treatments for menstrual migraine has been evaluated in studies described in Dowson 2005 [15], Tuchman 2006 [17], Cady 2011 [16], Landy 2004 [14], Massiou 2005 [18], Allais 2010 [20], Allais 2006 [21], Allais 2011 [22], Bartolini 2012 [23], Savi2011 [24], Silberstein 1999 [26], Newman 2001 [30], Mannix 2007 [31], Tuchman 2008 [32], Silberstein 2004 [33], Silberstein 2009 [34], Brande 2009 [35], and Sances 1990 [36]. However, there are studies with post-hoc analyses based on studies for chronic migraine. These studies include Mannix 2007 [11], Martin 2007 [12], Silberstein 2000 [13], Diamond 2008 [19], MacGregor 2022 [25], MacGregor 2018 [27], Igarashi 2022 [28], and Pavlovic 2020 [29].

### Outcomes

**Fig. 3:**
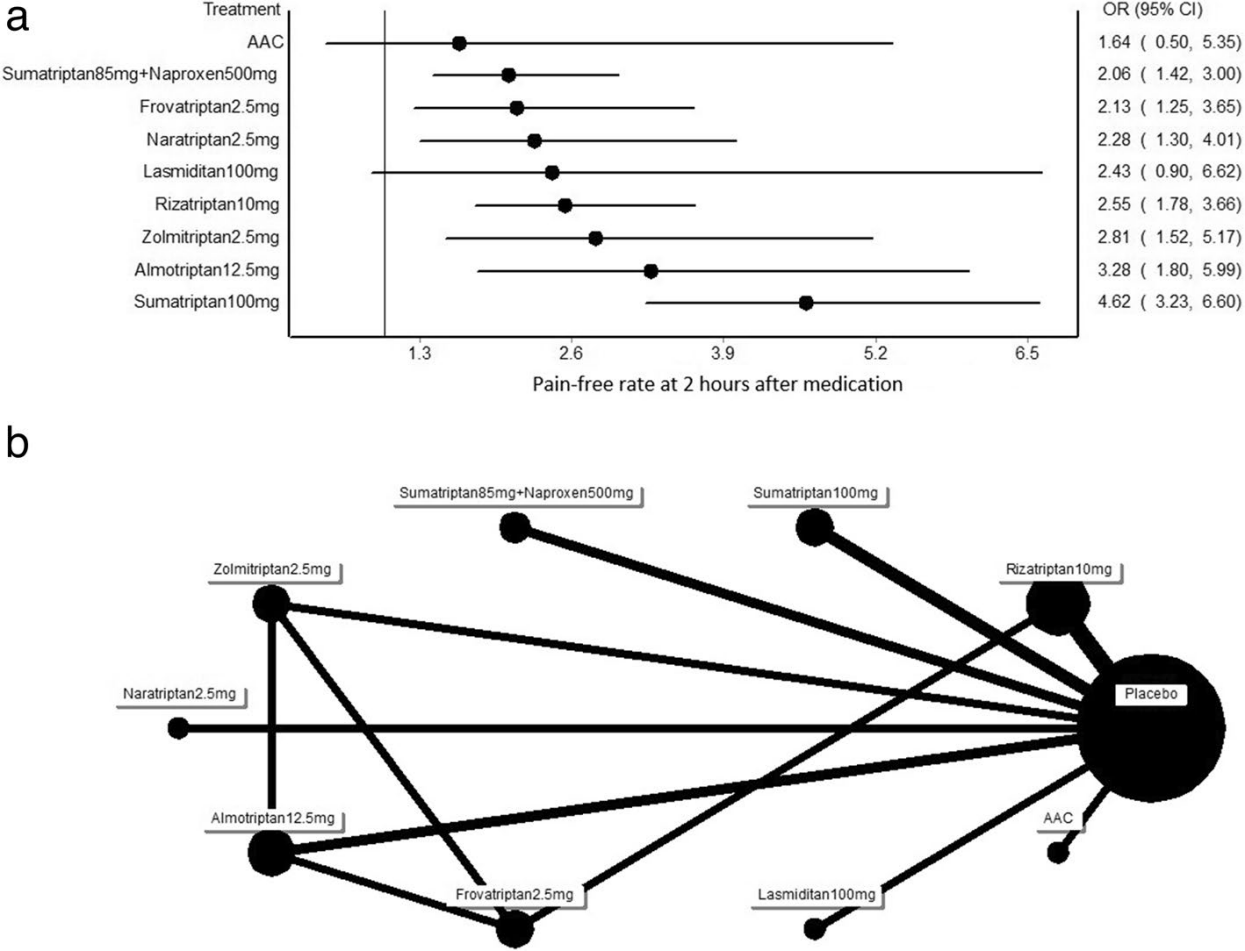
**a** Forest plot of the pain free rate at 2 h after using each triptan (pain killer) versus placebo. OR: odds ratio. **b**. Network diagram representing the eligible treatment comparisons for the multiple treatment comparison meta-analyses focusing on pain-relief response at 2 h after medication use. The size of nodes represents the number of patients, and line thickness represents the number of trials

**Fig. 4:**
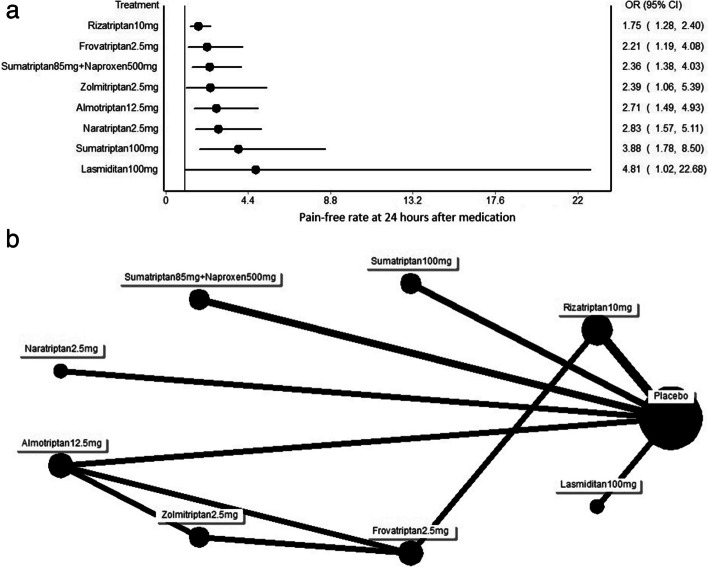
**a** Forest plot of the pain free rate at 24 h after using each triptan versus placebo. OR: odd ratio. **b** Network representing eligible treatment comparisons for the multiple treatment comparison meta-analyses focusing on pain relief responses at 24 h

Figures 3a and 4a illustrate the forest plots for the primary endpoints of each treatment versus placebo across multiple treatment comparisons. We selected headache relief at 2 h (Fig. 3a and b) and 24 h (Fig. 4a and b) as the outcomes of interest. All treatments were significantly more effective than placebo (defined as the 95% confidence interval precluding 1.00) at both 2 and 24 h. Sumatriptan had the highest OR among all treatments in the 2-h time period, with an OR value of 4.62 (95% CI: 3.23 to 6.60).

At 2 h after medication use, almotriptan had a higher OR (3.28; 95% CI: 1.80 to 5.99) than zolmitriptan (2.81; 95% CI: 1.52 to 5.17), rizatriptan at 10 mg (2.55; 95% CI: 1.78 to 3.66), lasmiditan at 100 mg (2.43; 95% CI: 0.90 to 6.62), naratriptan (2.28; 95% CI: 1.30 to 4.01), frovatriptan (2.13; 95% CI: 1.25 to 3.65), sumatriptan combined with naproxen (2.06; 95% CI: 1.42 to 3.00), and AAC (1.64; 95% CI: 0.50 to 5.35). Among all treatments studied over a 24-h period, lasmiditan had the highest OR (4.81; 95% CI: 1.02 to 22.68). Sumatriptan at 50 mg had a higher odds ratio (3.35; 95% CI: 1.88 to 6.00) than naratriptan (2.83; 95% CI: 1.57 to 5.11), almotriptan (2.71; 95% CI: 1.49 to 4.93), zolmitriptan (2.39; 95% CI: 1.06 to 5.39), sumatriptan combined with naproxen (2.36; 95% CI: 1.38 to 4.03), frovatriptan (2.21; 95% CI: 1.91 to 4.08), and rizatriptan at 10 mg (1.75; 95% CI: 1.28 to 2.40). Both 2 and 24 h after use, frovatriptan was less effective than all other triptans.

**Fig. 5:**
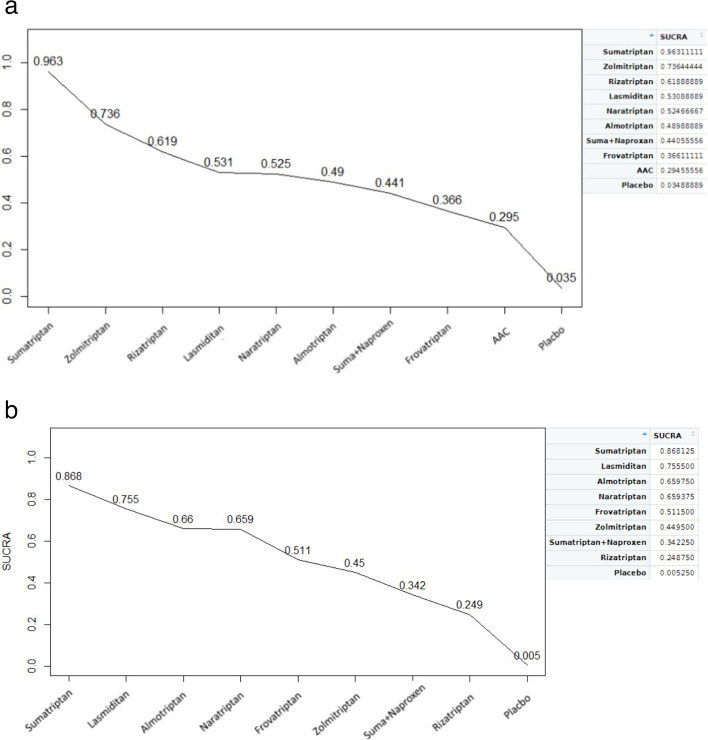
**a** Surface under the cumulative ranking (SUCRA) cluster plots of 2-h pain relief after treatment of menstrual migraine. **b** Surface under the cumulative ranking (SUCRA) cluster plots of 24-h pain relief after treatment of menstrual migraine

SUCRA provides a numerical representation of the overall ranking and returns a single number for each treatment that ranges from 0 to 100% (0 to 1). High SUCRA values indicate that a given medication is more likely to be ranked as one of the most effective treatments for MM. By contrast, low SUCRA values indicate that a given medication is more likely to be ranked as minimally effective. The treatments were ranked from high to low in terms of their effect on MM at 2 h after medication use in the following order: sumatriptan, zolmitriptan, rizatriptan, lasmiditan, naratriptan, almotriptan, sumatriptan combined with naproxen, frovatriptan, AAC, and placebo (Fig. 5a). The treatments were ranked from high to low in terms of pain relief at 24 h after medication use in the following order: sumatriptan, lasmiditan, almotriptan, naratriptan, frovatriptan, zolmitriptan, sumatriptan combined with naproxen, rizatriptan, and placebo (Fig. 5b).

SUCRA analysis revealed that sumatriptan was the most effective acute treatment for pain relief at both 2 h and 24 h. Lasmiditan, a novel 5-HT1F inhibitor, ranked second in the light of its effectiveness in pain relief 24 h after treatment.

**Fig. 6:**
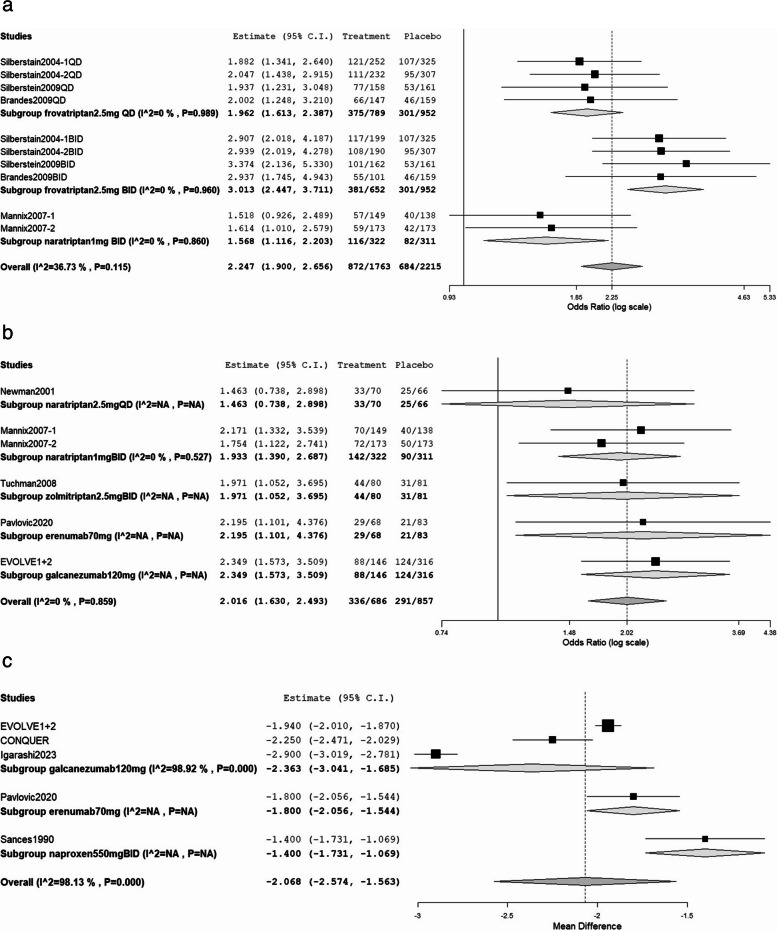
**a** Forest plot of no headache recurrence during the treatment period after prevention treatment. **b** Forest plot of patients who achieved ≥ 50% reduction from baseline in monthly migraine days after prevention treatment. **c** Forest plot of the decrease in the mean number of headache days per month after prevention treatment

In terms of preventing headache recurrence during treatment (Fig. 6a), frovatriptan 2.5 mg twice daily performed the best with an OR of 3.013 (95% CI: 2.447 to 3.711), followed by frovatriptan 2.5 mg once daily with OR of 1.962 (95% CI: 1.613 to 2.387). Although naratriptan 1 mg twice daily was less effective than both frovatriptan 2.5 mg once daily and twice daily, it was still efficient against headache recurrence with an OR of 1.568 (95% CI: 1.116 to 2.203). The overall results indicated significant effectiveness in preventing headache recurrence during the treatment period (OR: 2.247; 95% CI: 1.900 to 2.656). Figure 6b displays the forest plot for patients achieving ≥ 50% reduction in monthly migraine days, with galcanezumab being the most effective (OR: 2.349, 95% CI: 1.573 to 3.509), followed by erenumab (OR: 2.195, 95% CI: 1.101 to 4.376), zolmitriptan 2.5 mg twice daily (OR: 1.971, 95% CI: 1.052 to 3.695), naratriptan 1 mg twice daily (OR: 1.933, 95% CI: 1.390 to 2.687), and naratriptan 2.5 mg once daily (OR: 1.463, 95% CI: 0.738 to 2.898). The overall OR was 2.016 (95% CI: 1.630 to 2.493). Figure 6c presents a forest plot of the decrease in headache days per month. Galcanezumab yielded the most significant reduction (MDD: − 2.363; 95% CI: − 3.043 to − 1.683), whereas erenumab and naproxen had significant decreases with MDDs of − 2.100 (95% CI: − 2.345 to − 1.855) and − 1.400 (95% CI: − 1.727 to − 1.073), respectively. Overall, the MDD was − 2.28 (95% CI: − 2.620 to − 1.635).

### Risk of *bias* and inconsistency in studies

In the risk of bias analysis, we included only RCTs that had a low risk of bias by virtue of their well-described and rigorous randomization process. Figure 1 presents the risk of bias results; all considered studies had a low or unclear risk of bias overall.

The global inconsistency of the studies was calculated to be 44.22%, indicating moderate heterogeneity among the studies. This was attributed to the diversity of drugs compared within each study for the same endpoints. The local inconsistency analysis revealed mild to moderate heterogeneity among the studies.

## Discussion

Migraines' pathophysiology remains unclear; however, genetics, environment, and neurobiology are thought to play important roles. These factors could be related to changes in neural networks involved in the transmission of pain signals. When the central pain pathway is activated by either the cortex or brainstem, the trigeminal vascular system is stimulated and innervates cerebral blood vessels, causing the release of vascular inflammatory substances, such as CGRP, cytokines, and prostaglandins [37]. Estrogen affects vascular inflammatory substances at different doses to modulate the central pain pathway through multiple complex mechanisms [38]. As acute treatment, triptans are typically the first-line treatment for acute migraine and MM because of their high effectiveness in treating pain caused by these types of migraine.

Triptans are strong agonists of 5-HT1B/1D receptors; 5-HT1B receptors mediate the constriction of cranial vessels, and 5-HT1D receptors inhibit the release of sensory neuropeptides from perivascular trigeminal afferents. Trigeminal nucleus caudalis neurons receive input from the trigeminal nerve through 5-HT1B/5-HT1D receptors. Therefore, 5-HT1B/5-HT1D agonists may affect both the central and peripheral components of the trigeminal vascular system, and at least some of their effects may be mediated by the central nervous system [38]. Seven triptans are available in the market, namely sumatriptan, zolmitriptan, rizatriptan, naratriptan, almotriptan, frovatriptan, and eletriptan, and they bind to both 5-HT1B and 5-HT1D receptors [4]. We did not include eletriptan in this study because we did not find RCTs that tested this drug and met our inclusion criteria. The therapeutic effects of different triptans are determined on the basis of their potencies in activating 5-HT1B and 5-HT1D receptors. Coronary arteries also contain contractile 5-HT1B receptors. However, triptans do not activate 5-HT2A receptors, which are responsible for most contraction of coronary blood vessels by serotonergic signals, as much as ergotamine does. In patients without concomitant vascular disease, triptans are generally well-tolerated and safe [39]. Triptans may be categorized based on their onset of action: (1) sumatriptan, zolmitriptan, rizatriptan, and almotriptan have a faster onset of action and (2) naratriptan and frovatriptan have a slower onset of action [40]. However, how an individual responds to a specific triptan cannot be predicted. Failure to respond to one triptan does not necessarily indicate that another triptan will also be ineffective; therefore, if a patient’s response to a given triptan is unsatisfactory, a physician might advise them to use another triptan [41].

A standard treatment for MM has not been developed. This systematic review included 16 studies that analyzed the effects of sumatriptan, sumatriptan combined with naproxen, almotriptan, rizatriptan, naratriptan, zolmitriptan, frovatriptan, lasmiditan, and AAC on pain relief in the acute treatment of MM. Overall, all the treatments were more effective at relieving pain relative to placebos. All short-acting triptans—sumatriptan, almotriptan, zolmitriptan, and rizatriptan—exhibited better pain relief at 2 h. Sumatriptan resulted in better pain relief than did other triptans at 2 h. Sumatriptan was more effective in acute treatment than other triptans, probably because it has a short onset time of approximately 15 to 30 min. According to a previous study, the combination of sumatriptan 85 mg and naproxen 500 mg provided superior results to placebo in the treatment of menstrual migraine [42]. In our meta-analysis, both the forest plot analysis and the SUCRA statistical analysis indicated that this combination was not superior to sumatriptan 100 mg alone. A study has also demonstrated that this combination is not superior to sumatriptan alone [6]. Further double-blind RCTs may be needed to clarify the controversy.

Our results revealed that lasmiditan can also be effective against migraine attacks in the short term. Once lasmiditan is administered, the effects are typically observed within 1 or 2 h. It is sustained and effective in treating all aspects of acute migraine attacks throughout 24 to 48 h [9]. During our study, lasmiditan was most effective at 24 h among all compared treatments. In 2013, Hu et al. conducted a systematic review and meta-analysis that compared frovatriptan, naratriptan, and zolmitriptan at different doses with a placebo as a prophylactic treatment for MM. Frovatriptan and zolmitriptan were found to be more effective than naratriptan in preventing MM. Our data revealed the same result: frovatriptan was more effective in reducing headache recurrence during the treatment period than naratriptan. Furthermore, frovatriptan 2.5 mg twice daily reduces headache recurrence more effectively than 2.5 mg once a day. In another acute treatment study, frovatriptan and naratriptan were not as effective as other drugs for relieving headaches at 2 h, although their longer half-lives may cause them to be useful in some situations [4]. Frovatriptan has the longest elimination half-life (26 h) among all triptans and a moderate affinity for the 5-HT1F and 5-HT7 receptors [43]. Thus, frovatriptan is less effective than other triptans in treating acute pain at 2 and 24 h, but it is an excellent option for prophylactic treatment. In addition, frovatriptan was demonstrated to have the lowest recurrence rate and frovatriptan was more effective as a preventive treatment than naratriptan, which may be due to its longer half-life. Naratriptan is a triptan that is easily absorbed by the body, with approximately 70% oral bioavailability. Typically, the onset of action occurs within 1 to 2 h, and peak concentrations occur within 2 to 3 h after the ingestion of a 2.5-mg tablet of naratriptan. Moreover, naratriptan has a mean elimination half-life of 6 h. [44].

The results of our study indicated that both CGRP monoclonal abantibody, erenumab and galcanezumab, were more effective in preventing MM. As in Fig. 6b, more patients after treatment of CGRP monoclonal antibody monthly, erenumab or galcanezumab had ≧50% reduction of headache days than after treatment of daily dose of triptans for 6–7 days during the menstrual period. In Fig. 6c, both monthly galcanezumab and erenumab significantly reduced mean monthly headache days when compared with naproxen twice a day for 14 days. Besides, CGRP monoclonal antibodyg may have fewer adverse effects than traditional anti-migrainous medications (such as triptans) due to their more specific targeting of the migraine mechanism.

The sample sizes of erenumab and galcanezumab trials were smaller than those of other trials where CIs were wider, and more trials are needed to provide data to support the conclusion. There are limited trials on the effectiveness of other monoclonal antibodies that target CGRP (eptinezumab and fremanezumab) and gepants other than telcagepant for treating menstrual migraines. Despite the existence of a randomized controlled study evaluating telcagepant for the prevention of perimenstrual migraine, we did not include it in our analysis because it has been withdrawn from the market due to concerns regarding hepatotoxicity [45]. A real-world study suggests that fremanezumab relieves menstrual migraines [8]. Ubrogepant has also been shown to be effective as an acute treatment for perimenstrual migraine attacks in a post hoc analysis [46]. Available studies of fremanezumab and ubrogepant do not meet our inclusion criteria, their designs are not double-blind and contain no placebo.

## Conclusion

RCTs have generally demonstrated that all current treatments for migraine are effective in the acute treatment and prevention of MM. Sumatriptan is a more effective first-line treatment for acute migraine among all treatments. Other treatments may be considered for MM depending on how the patient responds to them. In addition, lasmiditan can be used as an acute treatment option. In addition, frovatriptan has been demonstrated to be the most effective preventive treatment for MM among all triptans available. Our findings indicate that galcanezumab and erenumab are excellent prophylactic treatments for MM. Thus, patients have more options for relieving MM.

## Supplementary Information

Supplementary Material 1

## Data Availability

All data supporting the findings of this study are availabe within the paper and its Supplementary information.

## Data availability

No datasets were generated or analysed during the current study.
